# A73 CANADIAN INFLAMMATORY BOWEL DISEASE MOBILE APPS: CURRENT LANDSCAPE AND NEEDS

**DOI:** 10.1093/jcag/gwab049.072

**Published:** 2022-02-21

**Authors:** B A Chiew, M Raman, P Tandon, R Panaccione, L Taylor

**Affiliations:** 1 University of Calgary Cumming School of Medicine, Calgary, AB, Canada; 2 University of Alberta Faculty of Medicine & Dentistry, Edmonton, AB, Canada

## Abstract

**Background:**

Evidence-based digital health applications (apps) offering comprehensive lifestyle therapies for inflammatory bowel disease (IBD) patients are limited in Canada.

**Aims:**

The aims of this study were to explore the Canadian IBD digital app landscape and review preliminary data from a recently launched digital app for IBD, LyfeMD. (www.lyfemd.ca).

**Methods:**

“IBD”, “Inflammatory bowel disease”, “UC”, “Ulcerative colitis”, “Crohns” and “Crohn’s disease (CD)” were searched by one team member (BC) on the App Store. Apps were included if they offered any type of lifestyle therapy, including education. The mobile application rating system (MARS) was used to evaluate each app and is a validated tool used to assess the quality of mobile health apps. For the LyfeMD app, 35 IBD users completed a baseline assessment survey to identify: 1) physical activity, sitting, and screen time, and; 2) stress, sleep, depression and anxiety. Eleven participants completed in-depth user experience evaluations after 4 weeks. Survey scores were calculated using published scoring protocols and descriptive data were prepared.

**Results:**

The LyfeMD and My IBD Care app scored highest on the MARS with a total score of 4.8/5. Of the other eight apps identified, scores ranged from 2.4 to 4.6 (overall mean=4.0). LyfeMD differentiated itself from other apps by providing lifestyle programs to improve nutrition, physical activity and mental health. Of the LyfeMD users, 74% had CD (median Harvey Bradshaw index=3.1, IQR=1.1–4.8) and 26% had ulcerative colitis (median partial mayo score=1.0, 0.5–6.0), 60% had a BMI ≥25 kg/m^2^, 57% were meeting 150 minute/week activity guidelines, 49% had high sitting time, 100% had high screen time, 69% had a moderate to high level of stress, 100% experienced sleep problems, 69% reported depression, and 49% reported anxiety. Eleven people completed the detailed user experience evaluations. They reported the app helped them identify behaviour changes to improve overall wellness; most often what they eat (64%), overall well-being (64%) and physical activity (46%).

**Conclusions:**

Two IBD apps available in Canada had a high MARS rating, however only the LyfeMD app offered comprehensive lifestyle therapies. The growing literature supports benefit for lifestyle therapies in IBD, and the LyfeMD app may be effective to identify areas amenable to lifestyle modification.

**Funding Agencies:**

Ascend, Alberta Innovates

**Table:** Table 1: MARS Scores

	IBD Healthline	My IBD Care: Crohn’s and Colitis	Gali Health	MyGut	myIBD	LyfeMD	Phyla Health	My Crohn’s and Colitis Support	AGA IBD Manager	Crohn’s Disease by AZoMedical
Engagement	3.00	4.75	4.00	3.80	2.00	4.80	3.60	3.60	3.00	2.40
Functionality	5.00	4.75	4.00	5.00	3.00	4.75	5.00	3.75	3.75	3.75
Aesthetics	4.33	5.00	5.00	5.00	1.67	5.00	4.33	3.33	4.33	4.67
Information Quality	3.86	4.71	3.29	4.43	2.86	4.71	3.86	3.71	4.29	4.00
Mean MARS	4.05	4.80	4.07	4.56	2.38	4.82	4.20	3.60	3.84	3.71

